# Perfluoroalkyl Substance (PFAS) Mixtures Drive Rheumatoid Arthritis Risk Through Immunosuppression: Integrating Epidemiology and Mechanistic Evidence

**DOI:** 10.3390/ijms26157518

**Published:** 2025-08-04

**Authors:** Yanming Lv, Chunlong Zhao, Yi Xiang, Wenhao Fu, Jiaqi Li, Fan Wang, Xueting Li

**Affiliations:** 1 Department of Toxicological Science, School of Public Health, Harbin Medical University, Harbin 150081, China; lvyanming2023@163.com (Y.L.); zhaochunlong023@163.com (C.Z.); xy1790942103@163.com (Y.X.); 18219773672@163.com (W.F.); 13314582001@163.com (J.L.); 2 Department of Epidemiology, School of Public Health, Harbin Medical University, Harbin 150081, China

**Keywords:** perfluoroalkyl substances (PFAS), rheumatoid arthritis, mixture exposure, NHANES, Bayesian kernel machine regression (BKMR), molecular docking

## Abstract

Perfluoroalkyl substances (PFASs) possess immunosuppressive properties. However, their association with rheumatoid arthritis (RA) risk remains inconclusive across epidemiological studies. This study integrates population-based and mechanistic evidence to clarify the relationship between PFAS exposure and RA. We analyzed 8743 U.S. adults from the NHANES (2005–2018), assessing individual and mixed exposures to PFOA, PFOS, PFNA, and PFHxS using multivariable logistic regression, Bayesian kernel machine regression, quantile g-computation, and weighted quantile sum models. Network toxicology and molecular docking were utilized to identify core targets mediating immune disruption. The results showed that elevated PFOA (OR = 1.63, 95% CI: 1.41–1.89), PFOS (OR = 1.41, 1.25–1.58), and PFNA (OR = 1.40, 1.20–1.63) levels significantly increased RA risk. Mixture analyses indicated a positive joint effect (WQS OR = 1.06, 1.02–1.10; qgcomp OR = 1.26, 1.16–1.38), with PFOA as the primary contributor. Stratified analyses revealed stronger effects in females (PFOA Q4 OR = 3.75, 2.36–5.97) and older adults (≥60 years). Core targets included EGFR, SRC, TP53, and CTNNB1. PFAS mixtures increase RA risk, dominated by PFOA and modulated by sex/age. These findings help reconcile prior contradictions by identifying key molecular targets and vulnerable subpopulations, supporting regulatory attention to PFAS mixture exposure.

## 1. Introduction

Polyfluoroalkyl substances (PFASs) are a class of synthetic chemicals that have been extensively utilized in industrial and consumer products [1,2]. These substances are distinguished by their remarkable environmental persistence and bioaccumulative potential [3,4]. These compounds resist degradation in ecosystems, contaminating water supplies, soil, and food chains, thereby posing long-term health risks to both wildlife and human populations [5]. It is a matter of concern that exposure to PFASs has been associated with immune system dysfunction across species, including diminished vaccine effectiveness in children, modified cytokine profiles, and heightened susceptibility to autoimmune diseases such as thyroiditis and lupus [6,7]. A growing body of evidence implicates various environmental chemicals in RA pathogenesis through disruption of immune homeostasis and inflammatory pathways. Occupational exposure to silica dust and cigarette smoke are established risk factors for RA, primarily mediated through activation of innate immune responses and generation of autoantigens [8,9]. Similarly, exposure to heavy metals like mercury has been linked to joint disorders, potentially through induction of oxidative stress and dysregulation of cytokine production [10]. Endocrine-disrupting chemicals (EDCs), prevalent environmental contaminants, have also been associated with altered immune function and increased susceptibility to autoimmune diseases, often involving modulation of hormonal signaling pathways that intersect with immune regulation [11,12]. Despite growing recognition of the ecological and toxicological impacts of PFASs, critical knowledge gaps persist regarding their combined effects on immune–inflammatory pathways. These PFAS mixtures, which are ubiquitous in environmental settings, may contribute to chronic disease pathogenesis. Particularly urgent is the need to elucidate their potential links to rheumatoid arthritis (RA) development through inflammatory mechanisms.

Rheumatoid arthritis (RA) has been defined as a systemic autoimmune disorder [13]. Its hallmarks are chronic inflammation of the joints and bone erosion, and it affects approximately 1% of the global population [14,15]. While genetic factors account for approximately 50% of the disease susceptibility, environmental triggers, including air pollutants, occupational hazards, and endocrine-disrupting chemicals, are increasingly implicated in the pathogenesis of RA [16,17]. Notably, the global rise in RA prevalence over the past three decades correlates with industrialization trends, suggesting a potential role of synthetic pollutants like PFASs [18,19]. Mechanistic studies reveal that PFASs disrupt innate and adaptive immunity by activating inflammatory pathways and inducing oxidative stress [20]. Recent studies have demonstrated that perfluorooctanoic acid (PFOA) has the capacity to elevate pro-inflammatory cytokines (TNF-α, IL-6) in murine models, while perfluorononanoic acid (PFNA) has been observed to induce thymic atrophy and persistent immunosuppression [21,22,23]. However, epidemiological evidence linking PFASs to RA remains sparse. A preceding occupational cohort has identified PFOA as a risk factor for RA [24]. The evidence from population-based studies is inconclusive, and the combined effects of PFAS mixtures have not yet been fully addressed.

Recent research has been chiefly concentrated on individual PFASs, thus neglecting the potential synergistic or antagonistic interactions that may occur in mixtures. Moreover, further research is required to elucidate the molecular mechanisms that underpin the association between PFAS exposure and RA pathogenesis, especially about immune–inflammatory cascades and genetic targets. To address these gaps, a cross-sectional study was conducted on 8743 U.S. adults from the NHANES (2005–2018). Advanced epidemiological models (Bayesian kernel machine regression and quantile g-computation) were integrated with network toxicology and molecular docking. Our objectives are threefold: (1) to evaluate single and mixed PFAS exposure effects on RA risk; (2) to identify vulnerable subpopulations based on age and sex; (3) to elucidate core molecular targets mediating PFAS-RA interactions, thereby bridging environmental exposure science with autoimmune disease mechanisms.

## 2. Results

### 2.1. Population Characteristics and PFAS Exposure

In the NHANES 2005–2018 cycles, 8743 adults aged between 20 and 85 years were included in the study, of whom 911 (11%) were diagnosed with RA. Table 1 summarizes the demographic characteristics of RA and non-RA participants. There were 564 female RA cases and 347 male RA cases. Statistically significant differences between RA and non-RA patients were observed in age, gender, race, marital status, BMI, smoking status, and diabetes prevalence. However, educational level, family PIR, and alcohol consumption were similar between the two groups.

**Figure 1:**
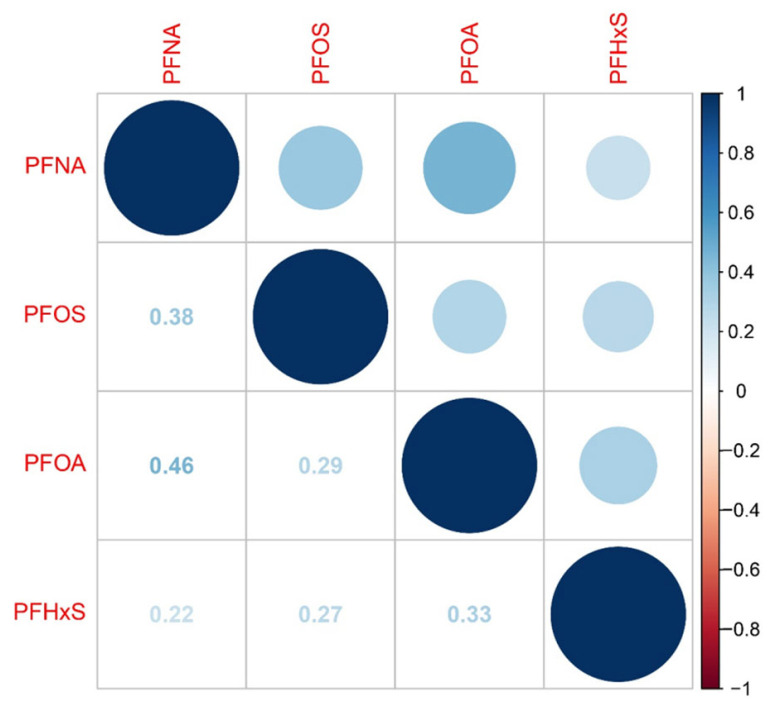
The Pearson correlation between PFAS metabolites after being log-transformed.

Table S1 presents the distribution of PFAS concentrations in serum samples. The detection rates of all PFOS metabolites surpassed 90%. Figure 1 illustrates the correlations among PFASs. PFOA exhibited a correlation coefficient of 0.46 with PFNA, 0.33 with PFHxS, and 0.29 with PFOS. PFOS showed a 0.38 correlation with PFNA and 0.27 with PFHxS. PFNA had a 0.22 correlation with PFHxS.

### 2.2. Logistic Regression Analysis of PFAS Metabolites Exposure and RA Risk

When PFAS metabolite concentrations were treated as continuous variables, PFOA, PFOS, and PFNA were associated with RA risk in the crude model (PFOA: 1.31 (1.18–1.46); PFOS: 1.17 (1.06–1.28); PFNA: 1.21 (1.08–1.37)) (Table 2). After adjusting for confounders in Model I and Model II, these correlations remained significant (Model I: PFOA: 1.60 (1.40–1.83), PFOS: 1.43 (1.28–1.60), and PFNA: 1.47 (1.27–1.70); Model II: PFOA: 1.63 (1.41–1.89), PFOS: 1.41 (1.25–1.58), and PFNA: 1.40 (1.20–1.63)). When PFAS concentrations were divided into quartiles, the Q4 group of PFOA, PFOS, and PFNA showed increased RA risk compared to the Q1 group. This pattern persisted after adjusting for confounders in Model I (PFOA: 3.24 (2.23–4.48); PFOS: 2.79 (2.01–3.87); PFNA: 2.24 (1.54–3.25)) and Model II (PFOA: 3.48 (2.46–4.92); PFOS: 2.65 (1.87–3.76); PFNA: 2.01 (1.38–2.93)).

Table S2 presents subgroup analyses by age. Among participants aged 40–60, RA incidence increased by 60%, 45%, and 38% per unit increase in PFOA, PFOS, and PFNA, respectively. The Q4 of PFOA (3.78 (2.06–6.90)), PFOS (2.84 (1.47–5.49)), and PFHxS (1.83 (1.00–3.34)) exposure was positively associated with RA incidence. Table S3 shows subgroup analyses by gender. Among female participants, RA incidence increased by 73%, 44%, and 52% per unit increase in PFOA, PFOS, and PFNA. The Q4 of PFOA (3.75 (2.36–5.97)), PFOS (2.51 (1.61–3.91)), and PFNA (2.45 (1.52–3.94)) exposure was positively associated with RA incidence.

### 2.3. WQS, Qgcomp, and BKMR Modeling for Joint Effect of PFAS Co-Exposure on RA Incidence

**Figure 2:**
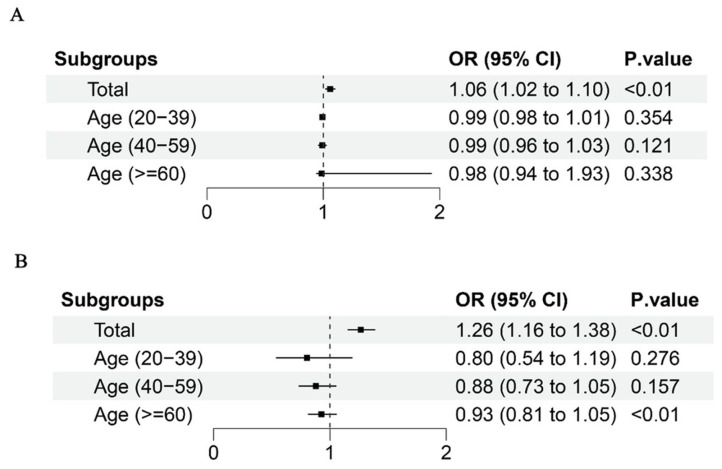
Odds ratios (95% CI) of RA associated with co-exposure to PFAS metabolites by WQS (**A**) and qgcomp (**B**) analyses in total population and subgroups. Models were adjusted for age, sex, race, education level, marital status, family PIR, BMI (kg/m^2^), smoking, drinking alcohol status, diabetes.

**Figure 3:**
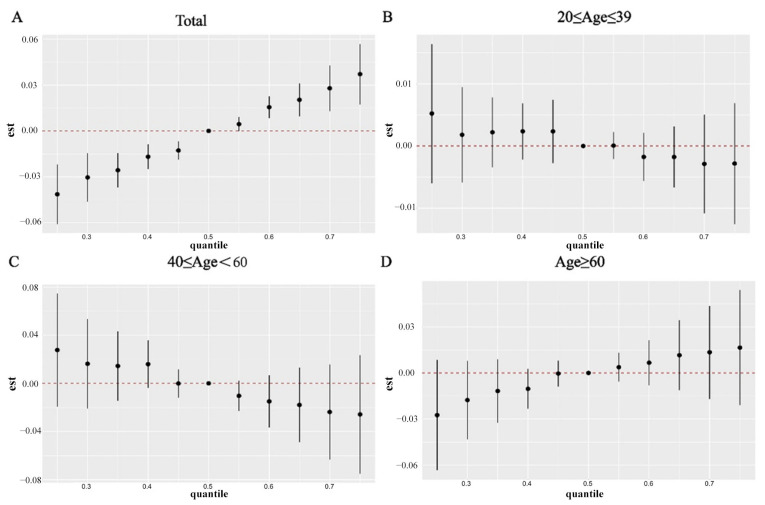
The joint effect of urinary PFAS metabolites on RA was estimated by BKMR models. (**A**) the total population; (**B**) 20 ≤ Age ≤ 39; (**C**) 40 ≤ Age ≤ 60; (**D**) Age ≥ 60.

**Figure 4:**
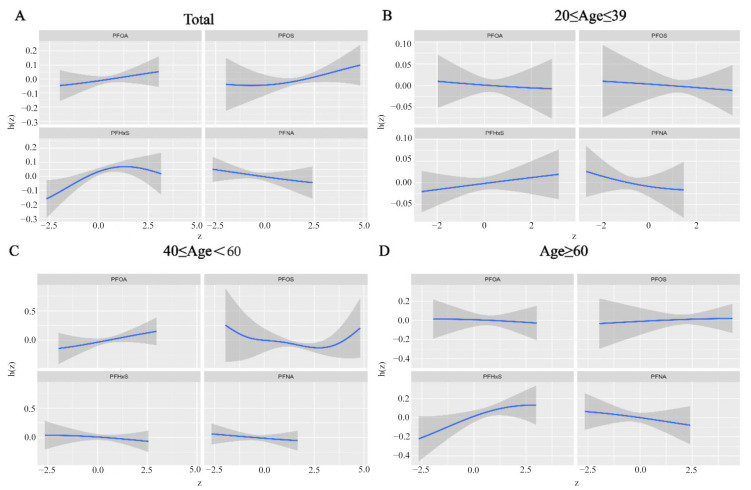
The univariate expose–response function (95% CI) between PFAS metabolites and RA was estimated using the BKMR model. (**A**) the total population; (**B**) 20 ≤ Age ≤ 39; (**C**) 40 ≤ Age ≤ 60; (**D**) Age ≥ 60.

To investigate the potential interactions among different PFAS mixtures and their collective impact on human health, we employed WQS, qgcomp, and BKMR analyses. The adjusted WQS model revealed a positive association between the WQS indices of the four PFAS metabolites and RA incidence (1.06 (1.02–1.10)) (Figure 2A). PFOA had the highest weight (0.67) in the mixture, followed by PFNA (0.19) and PFOS (0.12) (Figure S1). Similarly, qgcomp regression showed a positive link between PFAS co-exposure and RA incidence in all participants (1.26 (1.16–1.38)) (Figure 2B), with PFOA (weighted 0.566) being the dominant contributor (Figure S2). The BKMR model results indicated an overall increase in RA incidence in the total population as PFAS metabolite concentrations rose (Figure 3A). PFOA had the highest posterior inclusion probability (PIP = 0.724) among the PFAS metabolites (Table S4), and a positive dose–response relationship was observed between PFAS and RA incidence (Figure 4). We also examined the interactions between different chemicals (Figure S3). Key BKMR-derived statistics include a PIP of 0.724 for PFOA (Table S4) and stratified effect estimates, as shown in Figure S3.

### 2.4. Screening of PFAS- and RA-Related Target Genes

**Figure 5:**
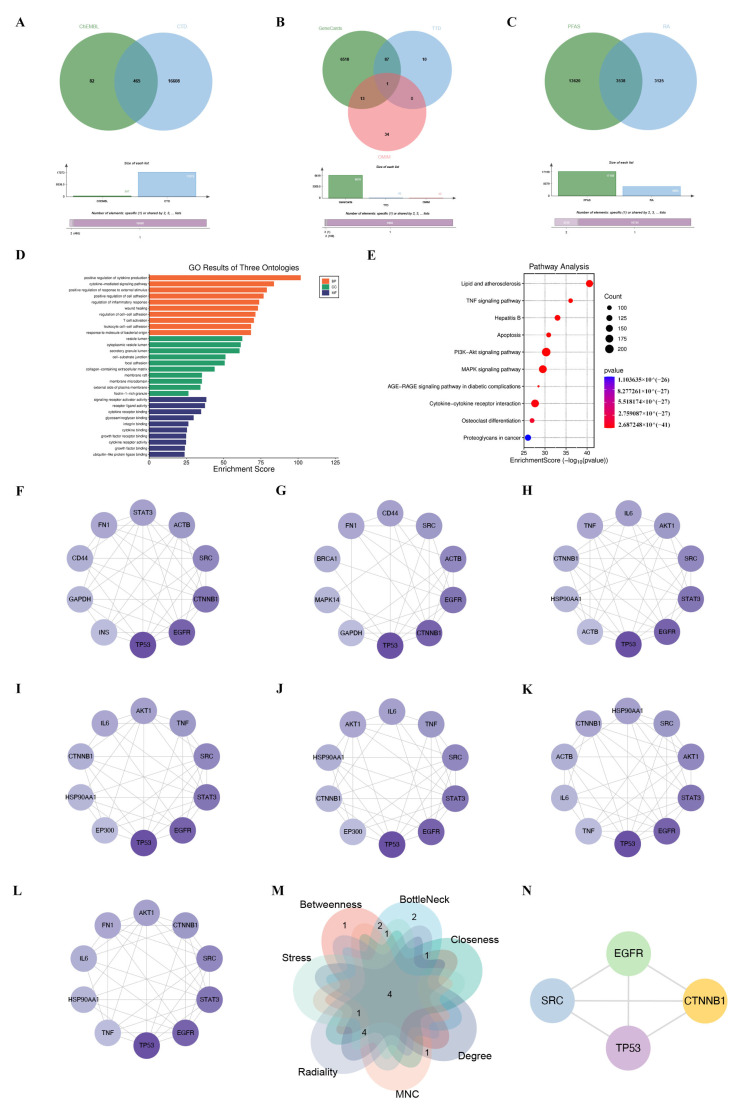
Network toxicology analysis. (**A**) Venn diagram of PFAS-related targets; (**B**) Venn diagram of RA-related targets; (**C**) Venn diagram of overlapping PFAS- and RA-related targets; (**D**) bar plot of Gene Ontology (GO) enrichment analysis; (**E**) bubble plot of Kyoto Encyclopedia of Genes and Genomes (KEGG) pathway enrichment analysis; top 10 hub targets calculated via (**F**) betweenness, (**G**) bottleneck, (**H**) closeness, (**I**) degree, (**J**) maximum neighborhood component (MNC), (**K**) radiality, and (**L**) stress; (**M**) Venn diagram illustrating target overlaps identified by seven topological algorithms; (**N**) protein–protein interaction (PPI) network of the four core targets.

**Figure 6:**
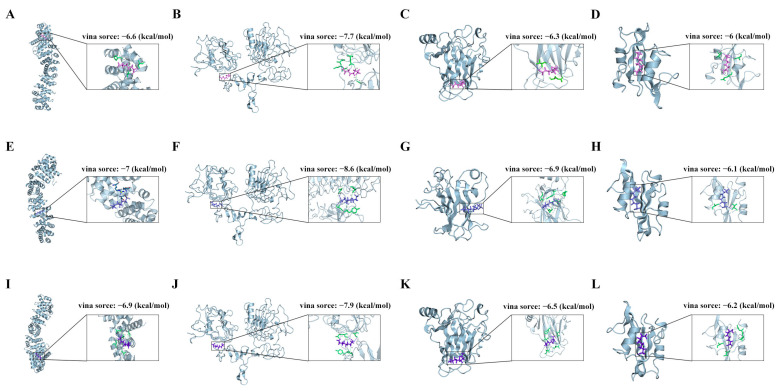
Molecular Docking. (**A**) PFOA-CTNNB1; (**B**) PFOA-EGFR; (**C**) PFOA-TP53; (**D**) PFOA-SRC; (**E**) PFOS-CTNNB1; (**F**) PFOS-EGFR; (**G**) PFOS-TP53; (**H**) PFOS-SRC; (**I**) PFNA-CTNNB1; (**J**) PFNA-EGFR; (**K**) PFNA-TP53; (**L**) PFNA-SRC.

Utilizing the comparative toxicogenomics database (CTD) and the ChEMBL database enabled the identification of 17,155 PFAS-related targets (Figure 5A). A total of 6663 RA-related targets were retrieved from GeneCards, the Therapeutic Target Database (TTD), and the Online Mendelian Inheritance in Man (OMIM) databases (Figure 5B). Among these, 3538 overlapping targets were selected as potential candidates underlying PFAS-induced RA pathogenesis (Figure 5C). GO enrichment analysis revealed significant associations of these targets with biological processes such as “positive regulation of cytokine production” and “cytokine-mediated signaling pathway,” as well as with cellular components like “vesicle lumen” and “secretory granule lumen,” and molecular functions including “signaling receptor activator activity” (Figure 5D). KEGG pathway analysis further emphasized their involvement in “signal transduction” and “cell growth and death” (Figure 5E). The construction of a protein–protein interaction (PPI) network was conducted using Cytoscape (version 3.10.3), and the top 10 hub targets were prioritized through the implementation of seven topological algorithms (Figure 5F,L). Subsequent Venn diagram analysis narrowed down the core targets to four key genes: The validation of the interactions between the proteins of interest (EGFR, SRC, TP53, and CTNNB1) was conducted using the STRING database (Figure 5N). Molecular docking analysis demonstrated robust binding interactions between PFASs and these core proteins, as visualized using PyMOL (Figure 6). Binding energies and interaction details are summarized in Table 3. PFOA exhibited the strongest binding to all targets, consistent with its dominant role in mixture toxicity.

## 3. Discussion

The present study synthesizes epidemiological and mechanistic evidence. This evidence shows that exposure to PFASs increases the risk of RA. PFOA, PFOS, and PFNA have been identified as the main contributors to this increased risk. The present findings build on previous occupational cohort studies by quantifying dose-dependent associations within the general population. Furthermore, the study emphasizes the importance of considering the synergistic effects of PFAS mixtures in real-world environmental exposure scenarios. We employed sophisticated modeling techniques (BKMR, WQS, and qgcomp). Our observations suggest that exposure to multiple PFAS compounds disrupts immune homeostasis, highlighting the limitations of current regulatory frameworks that evaluate chemicals in isolation. Given the environmental persistence of PFASs and their contamination of water supplies, soil, and food chains, these findings emphasize the urgent need to address mixture toxicity in ecological risk assessments. Contrary to recent NHANES analyses reporting inverse PFAS-RA associations [25], our study revealed positive dose–response relationships. Three methodological distinctions may explain this discrepancy: First, our cohort included occupational subgroups with serum PFOA >8.0 ng/mL (median: 4.7 ng/mL), exceeding background levels in Qiao et al. (median: 2.63 ng/mL). Second, mixture modeling quantified PFOA dominance (WQS weight = 0.67; PIP = 0.724), whereas BKMR in Qiao et al. showed no overall mixture pattern. Third, sex-stratified effects in females aligned directionally (OR = 3.75 vs. Qiao’s OR = 0.93) but diverged in magnitude due to exposure-level differences.

Notably, the increased vulnerability of females and older adults is consistent with the endocrine-disrupting and bioaccumulative properties of PFASs. Experimental evidence suggests that PFASs interfere with estrogen signaling, which could exacerbate autoimmune responses in women [3]. Meanwhile, age-related declines in metabolic detoxification could amplify the toxicity of PFASs in older populations [26]. Notable associations were found within these subgroups in the stratified analyses (Q4 PFOA OR = 3.75 in females), which may indicate biological vulnerability and differential environmental exposure patterns. Communities in close proximity to industrial sites or contaminated water sources frequently endure prolonged exposure to PFASs [27,28]. This situation is often exacerbated by the disproportionate impact on marginalized populations. The intersection of biological susceptibility and environmental injustice necessitates targeted biomonitoring and regulatory interventions to protect high-risk groups.

From a mechanistic perspective, PFAS-RA associations may involve inflammatory pathways [29]. These pathways are key regulators of cytokine production and immune cell activation [30]. Molecular docking was used to confirm the strong binding of PFASs to these targets. This suggests that PFASs disrupt immune signaling networks directly. The core targets identified (EGFR, SRC, TP53, and CTNNB1) are critically involved in RA pathogenesis through distinct mechanisms. EGFR activation promotes synovial fibroblast proliferation and inflammatory cytokine production, driving joint inflammation and erosion [31]. SRC kinase regulates immune cell activation and osteoclast-mediated bone resorption via RANKL signaling [32]. TP53 modulates oxidative stress responses and apoptosis in RA synovium; its dysregulation exacerbates tissue damage [33]. CTNNB1 participates in Wnt signaling that influences synovial hyperplasia and cartilage destruction [34,35]. Our molecular docking results demonstrate high-affinity binding of PFASs to these targets, suggesting PFASs may directly interfere with these pathways to promote RA development. These interactions are consistent with the findings of ecological studies. These studies indicate that exposure to PFASs in wildlife models results in thymic atrophy [22]. It also suppresses antiviral responses [36]. This compromises population resilience. Further research by the Network of Toxicology has linked PFASs to the activation of NF-κB and oxidative stress. These pathways are conserved across species and are involved in the pathogenesis of RA and broader ecological toxicity. PFASs trigger mitochondrial dysfunction, which interferes with energy metabolism and cell survival [37]. This phenomenon has significant similarities to human immune dysregulation.

Despite the advances achieved, the present study has its limitations. The cross-sectional design of the study means that causal inferences cannot be made. Additionally, self-reported RA diagnoses may introduce misclassification bias. Furthermore, while the present study focused on serum PFAS levels, future research should incorporate environmental sampling to elucidate exposure routes and bioaccumulation dynamics. The prolonged biological half-life of PFASs creates temporal complexities in assessments, requiring longitudinal cohorts with repeated biomonitoring. Additionally, while our mechanistic analysis is innovative, it would benefit from in vitro validation using environmentally relevant PFAS mixtures to better approximate real-world exposure scenarios. While our findings highlight PFAS mixtures as modifiable risk factors for RA, future multi-center studies across geographical regions are warranted to validate these associations in populations with varying genetic backgrounds and environmental exposure patterns. Such efforts would strengthen the global applicability of risk assessments.

In conclusion, our study demonstrates that PFAS exposure is a modifiable environmental risk factor for RA. The implications of this extend beyond human health. It also affects ecosystem stability. Therefore, regulatory action must prioritize restricting industrial discharges, phasing out non-essential PFAS uses, and enforcing stringent environmental limits on PFASs. International collaborations such as the Stockholm Convention should expand their scope. They should encompass PFAS mixtures and their transboundary contamination. At the same time, biomonitoring programs must integrate human and ecological data to identify vulnerable populations and species, ensuring holistic risk mitigation. This study highlights the need to view PFASs not just as chemical pollutants. It also emphasizes the importance of viewing them as catalysts of systemic, ecological, and immunological disruption. Future longitudinal cohort studies tracking PFAS exposure dynamics and RA incidence, combined with experimental validation using environmentally relevant PFAS mixtures, will be critical to confirm causality, clarify underlying mechanisms, and support evidence-based regulatory policies.

## 4. Materials and Methods

### 4.1. Study Population

The NHANES is a nationally representative, multistage, cross-sectional survey conducted by the National Center for Health Statistics (NCHS) on a biennial basis. The objective of the survey is to assess the health and nutritional status, in addition to potential risk factors, of the civilian population of the United States [38]. All NHANES projects have been approved by the NCHS Ethics Review Board, and participants provided written consent upon registration [39]. For this study, data were derived from the following six NHANES cycles: 2005–2006, 2009–2010, 2011–2012, 2013–2014, 2015–2016, and 2017–2018. These data, including demographic, examination, laboratory, and questionnaire data, were obtained from the official website and merged following the NHANES tutorial. Initially, 81,312 individuals participated in the NHANES study. After excluding participants who were uncertain about their RA status, those ineligible for PFAS measurement, those with missing covariate information, or those under the age of 20, a final analytic sample of 8743 participants was obtained, including 911 RA cases that completed both questionnaires and physical examinations.

### 4.2. Serum PFAS Measurement

Serum PFAS levels were quantified using online solid-phase extraction coupled with high-performance liquid chromatography–turbo ion spray ionization–tandem mass spectrometry (online SPE-HPLC-TIS-MS/MS). After standard phlebotomy, a minimum of 0.5 mL of serum was collected in standard containers and transferred to labeled polypropylene or polyethylene containers for frozen storage. Teflon^®^-coated materials were avoided. Specimens with signs of leakage, breakage, tampering, or insufficient volume were rejected. We measured four PFAS compounds: perfluorooctanoic acid (PFOA), perfluorooctane sulphonate (PFOS), perfluorohexane sulfonate (PFHxS), and perfluorononanoate (PFNA) [40]. For the 2013–2014 and 2015–2016 cycles, linear PFOA (n-PFOA), branched PFOA isomers (Sb-PFOA), linear PFOS (n-PFOS), and monomethyl branched PFOS isomers (Sm-PFOS) were measured. The total concentrations of these compounds were used to represent PFOA and PFOS levels.

### 4.3. Definition of RA

The status of RA was self-reported during personal interviews. The participants were firstly asked whether they had been informed by a doctor or other health professional that they had arthritis. In the event of a positive response, the participants were then asked to specify the type of arthritis from which they were suffering. Participants who confirmed having RA were classified as RA cases.

### 4.4. Collection of Covariates

In accordance with the findings of preceding studies, the following covariates were collated. The study’s demographic variables encompassed age (20–39 years, 40–59 years, ≥60 years), sex (male/female), race/ethnicity (Mexican American, non-Hispanic white, non-Hispanic black, other Hispanic, other race/multiracial), education level (high school, lower than high school, greater than high school), marital status (married/living with partner, widowed/divorced/separated, never married), and family poverty income ratio (PIR) (≤1). The following factors were considered: body mass index (BMI) (under 25, 25–30, over 30 kg/m^2^), smoking status, alcohol consumption status, and diabetes status [41].

### 4.5. Screening of Potential Molecular Targets

The prediction of potential PFAS targets was conducted by means of the utilization of the Comparative Toxicogenomics Database (CTD) and the ChEMBL database. RA-related gene targets were retrieved from GeneCards, the Therapeutic Target Database (TTD), and the Online Mendelian Inheritance in Man (OMIM) databases. Venn diagrams were plotted to identify overlapping targets that may link PFAS exposure to RA. Gene Ontology (GO) and Kyoto Encyclopedia of Genes and Genomes (KEGG) pathway enrichment analyses of these overlapping targets were performed using the R package (version 4.1.1) “ClusterProfiler”. A protein–protein interaction (PPI) network was constructed using Cytoscape (Version 3.10.3), and core targets were further screened based on the following seven topological algorithms: maximum neighborhood component (MNC), stress, degree, closeness, betweenness, bottleneck, and radiality.

### 4.6. Molecular Docking

The protein structures of the core targets were retrieved from the Protein Data Bank (PDB) database, and the PFAS structural data were obtained from the PubChem database. The energy minimization of PFOA, PFOS and PFNA molecules was performed using Chem3D 22.0.0. Target protein structures were preprocessed, and molecular docking was conducted using AutoDock Vina. Binding interactions were analyzed using the Protein–Ligand Interaction Profiler (PLIP), and results were visualized with PyMOL (Version 2.5.4). Docking scores were calculated using AutoDock Vina’s affinity scoring function, with lower values indicating stronger binding.

### 4.7. Statistical Analysis

To obtain nationally representative estimates, we accounted for the complex multi-stage sampling design of the NHANES. Baseline characteristics of participants by RA status were compared using Kruskal–Wallis tests and *t*-tests. Continuous variables were presented as mean ± SD, and the number of categorical variables as n (%). Since PFAS measurements were only available for one-third subsamples in each NHANES cycle, we used specific subsample A weights and divided them by 6 to construct combined weights for the six cycles. All PFAS variables were natural log-transformed and divided into quartiles (Q1–Q4). Pearson correlation analysis assessed correlations between PFASs. Weighted logistic regression models examined PFAS-RA associations, comparing Q2–Q4 to Q1. OR with 95% CIs were calculated using crude and multivariable models adjusting for age, sex, and other covariates.

To evaluate the joint effect of PFAS mixtures, we used Bayesian kernel machine regression (BKMR) via the “bkmr” R package. It estimates multivariable exposure–response functions while adjusting for covariates [42]. The combined effect was assessed by comparing RA estimates per 5% increase/decrease in PFAS mixture median concentrations. Posterior inclusion probability (PIP) determined the importance of PFAS mixtures. WQS regression via the “gWQS” R package assessed the overall PFAS mixture impact and individual PFAS contributions [43]. Data were split into training (40%) and validation (60%) sets. The qgcomp model via the “qgcomp” R package was also applied, assigning positive/negative weights to compounds with directional weight sums of 1. All analyses were performed in R (version 4.1.1), with significance set at *p* < 0.05.

## Figures and Tables

**Table 1:** Characteristics of participants with available data on PFASs and RA in the NHANES 2005–2018 cycles.

Characteristic	Overall, N = 8743 (100%)	Non-RA, N = 7832 (89%)	RA, N = 911 (11%)	*p*-Value
**Age, N (%)**				<0.001
Young (20–39)	3315 (39.89%)	3257 (43.77%)	58 (7.21%)	
Middle (40–59)	2648 (35.49%)	2392 (35.82%)	256 (32.75%)	
Old (≥60)	2780 (24.61%)	2183 (20.42%)	597 (60.03%)	
**Gender, N (%)**				<0.001
Female	4316 (49.29%)	3752 (47.93%)	564 (60.73%)	
Male	4427 (50.71%)	4080 (52.07%)	347 (39.27%)	
**Race, N (%)**				<0.001
Mexican American	1456 (8.55%)	1368 (9.19%)	88 (3.15%)	
Non-Hispanic Black	1789 (10.33%)	1615 (10.58%)	174 (8.25%)	
Non-Hispanic White	3848 (68.97%)	3341 (67.64%)	507 (80.17%)	
Other Hispanic	753 (5.36%)	684 (5.61%)	69 (3.30%)	
Other Race	897 (6.79%)	824 (6.98%)	73 (5.13%)	
**Educational level, N (%)**				0.4
Greater than high school	4762 (62.66%)	4284 (62.82%)	478 (61.37%)	
High school	1963 (22.92%)	1751 (22.68%)	212 (24.99%)	
Lower than high school	2018 (14.41%)	1797 (14.51%)	221 (13.63%)	
**Marital status, N (%)**				<0.001
Married/living with partner	5347 (64.22%)	4825 (64.18%)	522 (64.56%)	
Never married	1714 (19.42%)	1642 (21.03%)	72 (5.88%)	
Widowed/divorced/separated	1682 (16.36%)	1365 (14.79%)	317 (29.56%)	
**Family PIR, N (%)**				0.6
<1.30	2603 (19.93%)	2327 (20.06%)	276 (18.85%)	
>3.50	2821 (44.49%)	2545 (44.54%)	276 (44.04%)	
1.30–3.5	3319 (35.58%)	2960 (35.39%)	359 (37.11%)	
**BMI (kg/m^2^), N (%)**				<0.001
<25	2699 (32.04%)	2495 (33.21%)	204 (22.18%)	
>30	3063 (33.61%)	2609 (31.89%)	454 (48.12%)	
25–30	2981 (34.35%)	2728 (34.91%)	253 (29.70%)	
**Smoking, N (%)**	3778 (43.41%)	3299 (42.41%)	479 (51.89%)	<0.001
**Drinking alcohol status, N (%)**	6561 (80.73%)	5885 (80.64%)	676 (81.45%)	0.6
**Diabetes, N (%)**	1059 (9.41%)	813 (7.83%)	246 (22.77%)	<0.001

BMI, body mass index; N, number of subjects; NHANES, National Health and Nutrition Examination Survey; PIR, poverty income ratio; RA, rheumatoid arthritis.

**Table 2:** Associations of PFAS metabolites with RA risk in the NHANES 2005–2018 cycles.

	Crude Model		Model I		Model II	
	Crude OR (95%CI)	*p*-Value	Adjusted OR (95%CI)	*p*-Value	Adjusted OR (95%CI)	*P* for Trend
PFOA						
Continuous	1.31 (1.18–1.46)	<0.001	1.60 (1.40–1.83)	<0.001	1.63 (1.41–1.89)	<0.001
Q1	Reference		Reference		Reference	
Q2	1.10 (0.83–1.44)		1.38 (1.04–1.82)		1.51 (1.14–2.00)	
Q3	1.04 (0.80–1.35)	<0.001	1.48 (1.10–1.98)	<0.001	1.50 (1.11–2.02)	<0.001
Q4	2.21 (1.68–2.91)		3.24 (2.23–4.48)		3.48 (2.46–4.92)	
PFOS						
Continuous	1.17 (1.06–1.28)	<0.001	1.43 (1.28–1.60)	<0.001	1.41 (1.25–1.58)	<0.001
Q1	Reference		Reference		Reference	
Q2	1.05 (0.81–1.37)		1.40 (1.04–1.88)		1.36 (1.00–1.83)	
Q3	1.21 (0.91–1.61)	0.003	1.90 (1.41–2.57)	<0.001	1.86 (1.37–2.51)	<0.001
Q4	1.61 (1.20–2.16)		2.79 (2.01–3.87)		2.65 (1.87–3.76)	
PFHxS						
Continuous	0.99 (0.90–1.09)	0.8	1.10 (0.99–1.22)	0.085	1.11 (0.99–1.24)	0.079
Q1	Reference		Reference		Reference	
Q2	0.71 (0.54–0.93)		0.84 (0.62–1.15)		0.89 (0.65–1.23)	
Q3	0.80 (0.61–1.06)	0.020	1.13 (0.84–1.52)	0.066	1.17 (0.86–1.59)	0.10
Q4	0.97 (0.72–1.31)		1.27 (0.92–1.76)		1.33 (0.94–1.87)	
PFNA						
Continuous	1.21 (1.08–1.37)	<0.001	1.47 (1.27–1.70)	<0.001	1.40 (1.20–1.63)	<0.001
Q1	Reference		Reference		Reference	
Q2	0.92 (0.68–1.26)		1.18 (0.85–1.63)		1.12 (0.80–1.15)	
Q3	1.24 (0.89–1.73)	0.004	1.70 (1.18–2.45)	<0.001	1.65 (1.15–2.39)	<0.001
Q4	1.49 (1.08–2.06)		2.24 (1.54–3.25)		2.01 (1.38–2.93)	

**Table 3:** The vina source of molecular docking.

Compounds	Targets	PDB ID	Vina Source (kcal/mol)
PFOA	CTNNB1	1jpw	−6.6
	EGFR	1ivo	−7.7
	TP53	1kzy	−6.3
	SRC	1a07	−6.0
PFOS	CTNNB1	1jpw	−7.0
	EGFR	1ivo	−8.6
	TP53	1kzy	−6.9
	SRC	1a07	−6.1
PFNA	CTNNB1	1jpw	−6.9
	EGFR	1ivo	−7.9
	TP53	1kzy	−6.5
	SRC	1a07	−6.2

The binding energy value reflects the strength of the interaction between the small molecule and the macromolecule; the more negative the binding energy value, the stronger the binding force and the higher the stability of the complex.

## Data Availability

All data are open access and available for download at url: https://www.cdc.gov/nchs/nhanes (accessed on 28 December 2024).

## Supplementary Materials

The following supporting information can be downloaded at: https://www.mdpi.com/article/10.3390/ijms26157518/s1.
