# Supplementary material for: Perfluoroalkyl Substance (PFAS) Mixtures Drive Rheumatoid Arthritis Risk Through Immunosuppression: Integrating Epidemiology and Mechanistic Evidence
Source: Int J Mol Sci. 2025 Aug 4;26(15):7518. doi: 10.3390/ijms26157518 (PMC12347686; doi:10.3390/ijms26157518)
Supplement: Supplementary file 1 [file ijms-26-07518-s001.zip › ijms-3737940-supplementary.pdf]

Perfluoroalkyl Substances (PFAS) Mixtures Drive Rheumatoid Arthritis Risk through Immunosuppression: Integrating Epidemiology and Mechanistic Evidence

Yanming Lv [1,a], Chunlong Zhao [1,a], Yi Xiang [1], Jiaqi Li [1], Wenhao Fu [1], Fan Wang [2,*], Xueting Li [1,*]

[1] Department of Toxicological Science, School of Public Health, Harbin Medical University, Harbin 150081, China.

[2] Department of Epidemiology, School of Public Health, Harbin Medical University, Harbin, Heilongjiang Province, China.

[a] These authors contributed equally to this article.

*Correspondence should be addressed to Xueting Li and Fan Wang: lxting@hrbmu.edu.cn; yifan.701@163.com.

Table S1. The distributions of four PFAS metabolites in urine in the NHANES 2005-2018 cycles

PFAS (ng/mL)	Detection rates (%)	Mean	Minimum	P25	P50	P75	Maximum
PFOA (ng/mL)	99.8%	3.10	0.07	1.47	2.40	3.90	104
PFOS (ng/mL)	100%	12.23	0.14	4.20	8.10	14.70	1403
PFHxS (ng/mL)	98.6%	2.13	0.07	0.80	1.40	2.50	81.60
PFNA (ng/mL)	98.3%	1.20	0.06	0.5	0.9	1.40	25.75

Table S2. Associations of blood PFAS with RA risk stratified by age, NHANES, 2005-2018.

PFAS	subgroup	Continuous	Q1	Q2	Q3	Q4	*p* for trend
		OR (95%CI)		OR (95%CI)	OR (95%CI)	OR (95%CI)	
PFOA	20≤Age<40	0.62 (0.41-0.92)	reference	0.43 (0.16-1.11)	0.63 (0.25-1.56)	0.18 (0.06-0.53)	<0.001
	40≤Age<60	1.60 (1.23-2.09)	reference	1.65 (1.06-2.58)	1.34 (0.85-2.13)	3.78 (2.06-6.90)	<0.001
	Age≥60	0.60 (0.49-0.73)	reference	0.84 (0.56-1.26)	0.57 (0.35-0.92)	0.33 (0.21-0.52)	<0.001
PFOS	20≤Age<40	0.72 (0.53-0.97)	reference	0.91 (0.39-2.11)	0.52 (0.22-1.25)	0.54 (0.17-1.67)	<0.001
	40≤Age<60	1.45 (1.15-1.83)	reference	1.58 (0.93-2.67)	2.20 (1.16-4.16)	2.84 (1.47-5.49)	0.012
	Age≥60	0.73 (0.63-0.85)	reference	0.86 (0.61-1.21)	0.81 (0.55-1.20)	0.41 (0.28-0.60)	<0.001
PFHxS	20≤Age<40	0.84 (0.62-1.12)	reference	0.92 (0.40-2.14)	0.84 (0.37-1.97)	0.57 (0.20-1.60)	<0.001
	40≤Age<60	1.14 (0.92-1.41)	reference	1.03 (0.63-1.67)	1.48 (0.90-2.44)	1.83 (1.00-3.34)	<0.001
	Age≥60	0.94 (0.39-0.70)	reference	0.74 (0.50-1.11)	0.76 (0.48-1.20)	0.72 (0.48-1.06)	0.3
PFNA	20≤Age<40	0.57 (0.39-0.82)	reference	0.55 (0.21-1.42)	0.80 (0.33-1.96)	0.16 (0.05-0.47)	<0.001
	40≤Age<60	1.38 (1.03-1.83)	reference	1.43 (0.83-2.47)	1.53 (0.88-2.77)	1.77 (0.98-3.22)	<0.001
	Age≥60	0.74 (0.62-0.88)	reference	0.84 (0.59-1.20)	0.63 (0.43-0.91)	0.52 (0.36-0.75)	0.002

Models were adjusted for sex, race, education level, marital status, family PIR, BMI (kg/m2), smoking, drinking alcohol status, diabetes. Continuous, Ln-transformed concentration of PFAS; Q, quartile.

Table S3. Associations of blood PFAS with RA risk stratified by sex, NHANES, 2005-2018.

PFAS	subgroup	Continuous	Q1	Q2	Q3	Q4	*p* for trend
		OR (95%CI)		OR (95%CI)	OR (95%CI)	OR (95%CI)	
PFOA	Female	1.73 (1.43-2.07)	reference	1.85 (1.24-2.75)	1.73 (1.12-2.69)	3.75 (2.36-5.97)	<0.001
	Male	0.62 (0.50-0.77)	reference	1.03 (0.61-1.73)	0.67 (0.38-1.17)	0.39 (0.22-0.69)	<0.001
PFOS	Female	1.44 (1.24-1.67)	reference	1.42 (0.95-2.11)	1.65 (1.10-2.48)	2.51 (1.61-3.91)	<0.001
	Male	0.70 (0.57-0.87)	reference	0.63 (0.36-1.10)	0.58 (0.32-1.05)	0.37 (0.21-0.67)	0.005
PFHxS	Female	1.16 (1.00-1.35)	reference	0.91 (0.50-1.64)	1.08 (0.66-1.75)	1.54 (0.88-2.70)	0.062
	Male	0.92 (0.52-1.13)	reference	0.76 (0.46-1.26)	0.68 (0.39-1.19)	0.67 (0.39-1.14)	0.4
PFNA	Female	1.52 (1.24-1.85)	reference	1.02 (0.64-1.63)	1.36 (0.84-2.20)	2.45 (1.52-3.94)	<0.001
	Male	0.78 (0.61-0.99)	reference	1.10 (0.66-1.85)	0.79 (0.46-1.34)	0.75 (0.44-1.30)	0.4

Models were adjusted for age, race, education level, marital status, family PIR, BMI (kg/m^2), smoking, drinking alcohol status, diabetes. Continuous, Ln-transformed concentration of PFAS; Q, quartile.

Table S4. The posteriori inclusion probability of single PFAS metabolites.

PFAS	PIP
PFOA	0.724
PFOS	0.714
PFNA	0.572
PFHxS	0.482

PIP, posteriori inclusion probability.

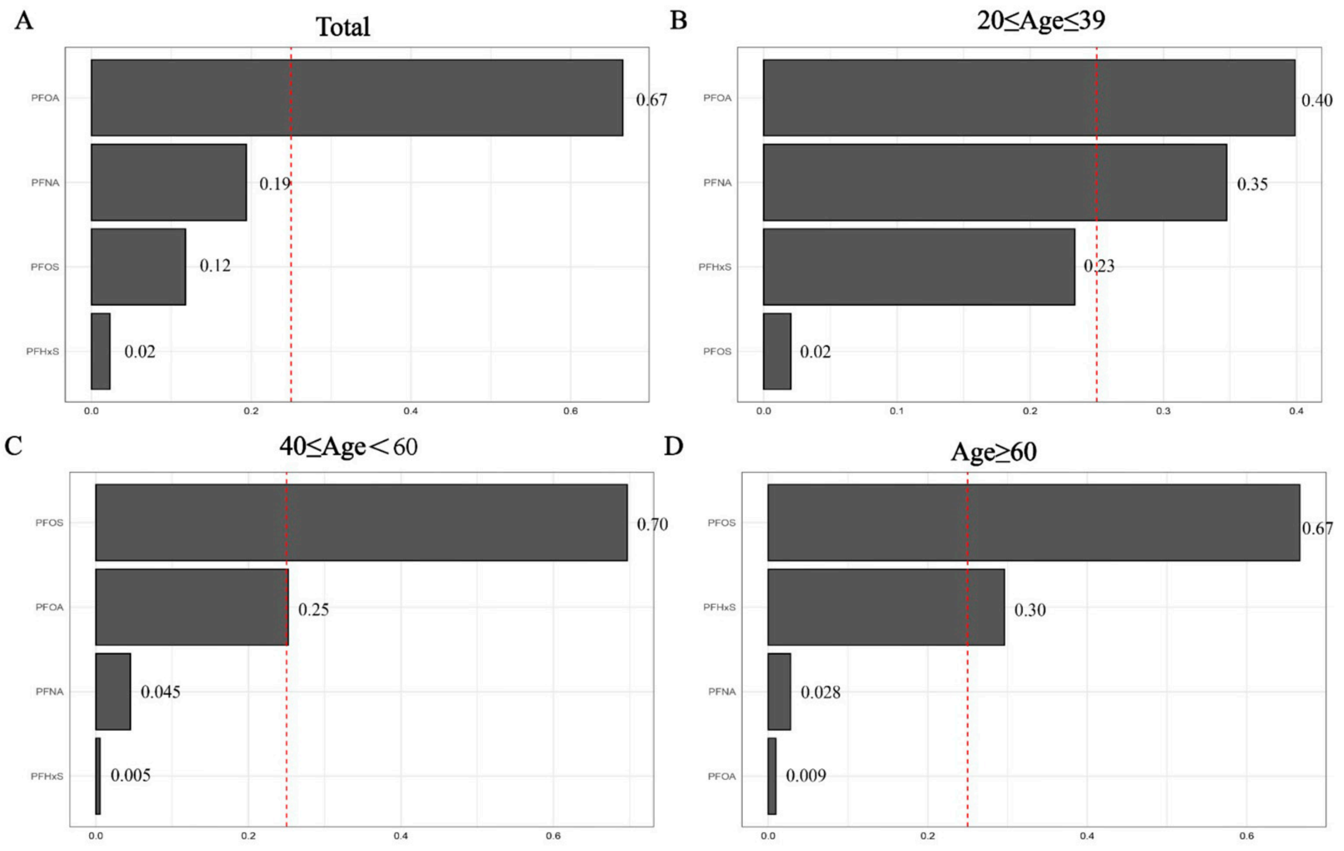

Figure S1. Estimated weights of PFAS metabolites for RA by WQS models**.**

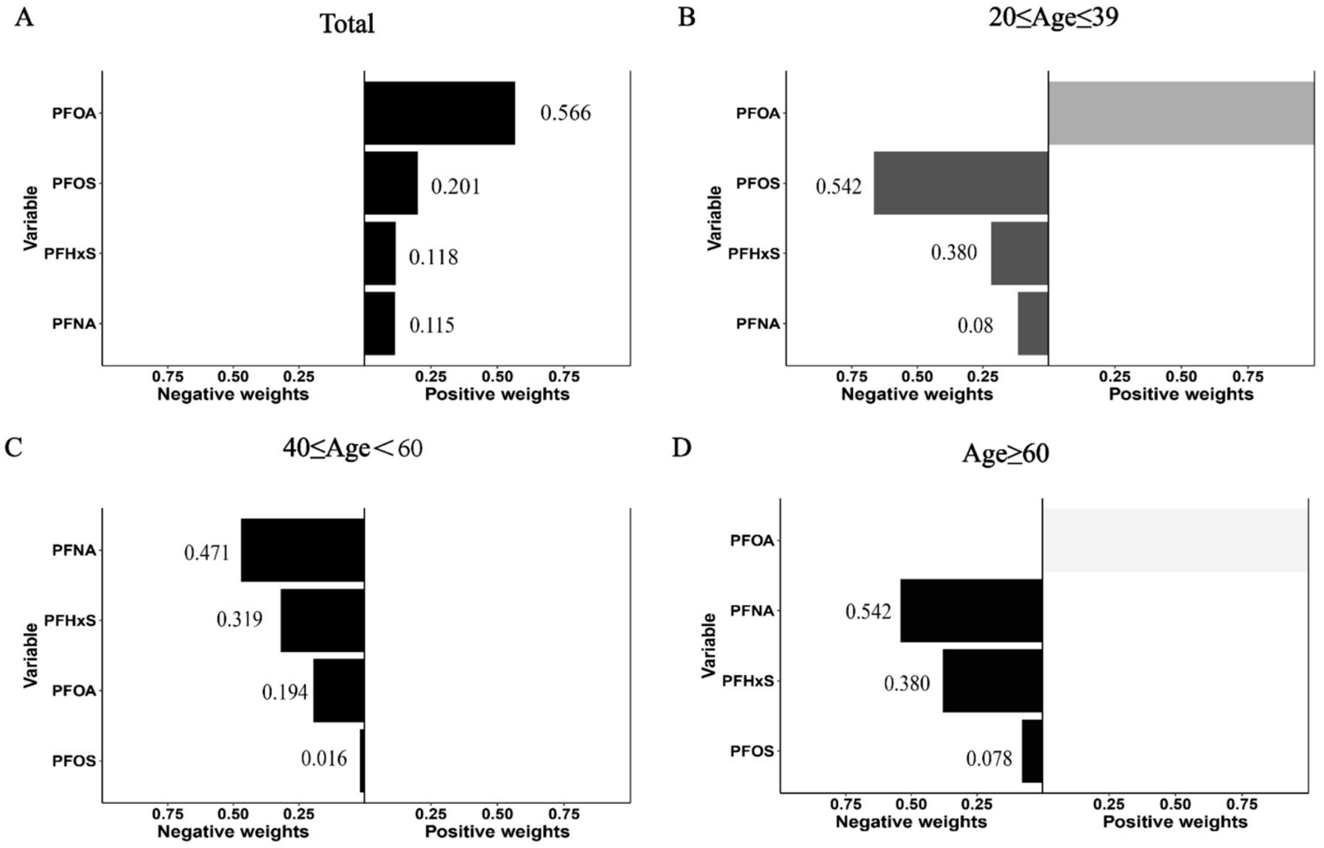

\

Figure S2. The positive and negative weights of PFAS metabolites for RA by qgcomp model**.**

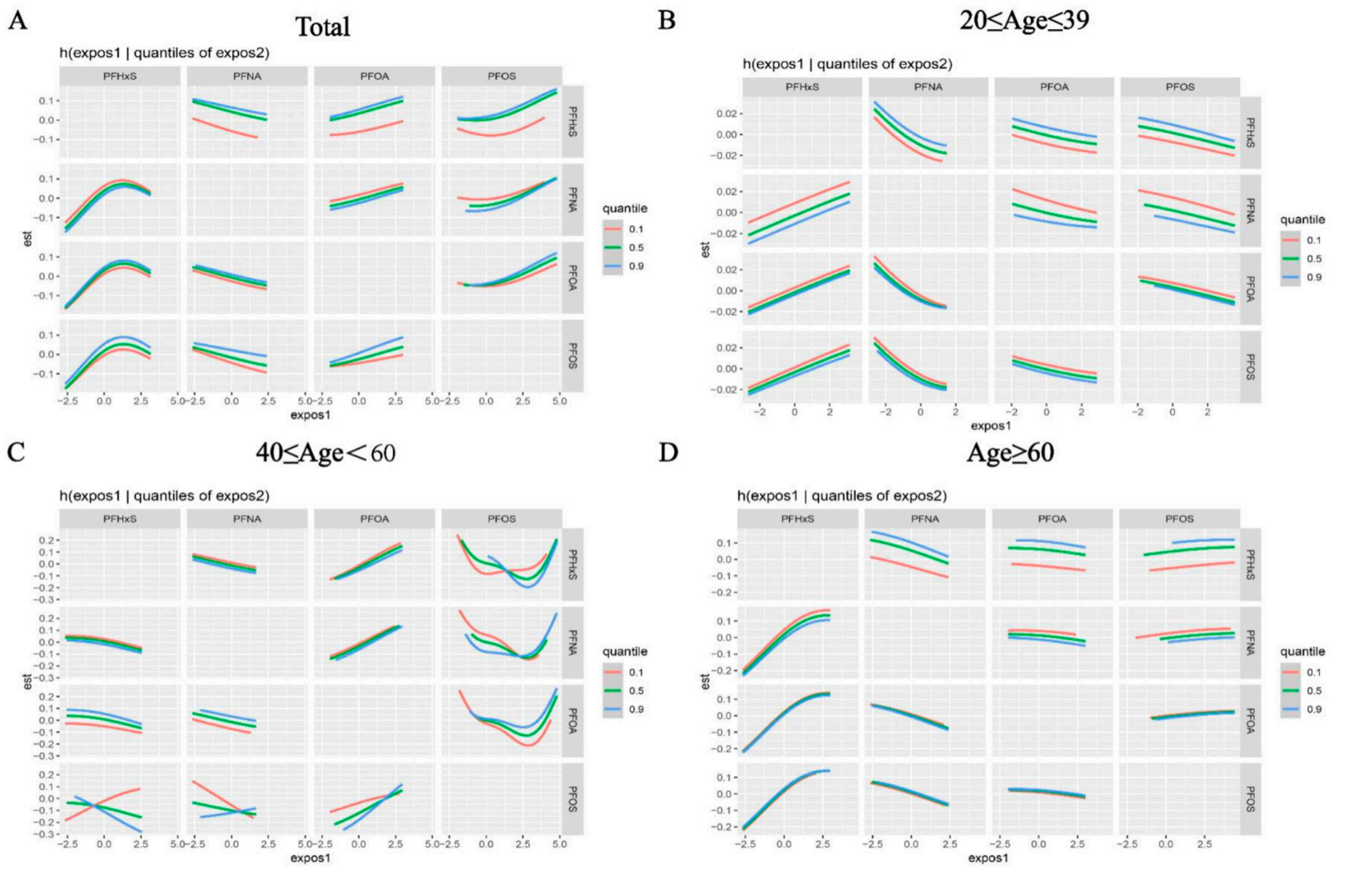

Figure S3. Bivariate exposure-response functions for RA and each of the PFAS when exposure chemicals were at their 25th (red line), 50th (green line), and 75th (blue line) percentile, and other chemicals were fixed at their median levels.
